# Cellular immune responses and changes in VL after a Dendritic Cells (DC)-based therapeutic vaccine in cART treated chronic HIV-infected patients with CD4 T cells above 450/mm

**DOI:** 10.1186/1742-4690-9-S2-O42

**Published:** 2012-09-13

**Authors:** F García, AC Guardo, M Maleno, L Papagno, M Bargalló, N Climent, B Autran, J Gatell, T Gallart, M Plana

**Affiliations:** 1 INSERM U543 - Université Paris VI Pierre et Marie Curie, ORVACS, Paris, France; 2 IDIBAPS - Hospital Clinic, Barcelona, Spain

## Background

We have performed a blinded placebo-controlled study immunizing antiretroviral (cART) treated chronically HIV-1 infected patients with autologous Myeloid-Derived Dendritic Cells (MD-DCs) pulsed with heat inactivated autologous HIV-1.

## Methods

36 patients with CD4+ >450 cells/mm3 were randomized (2:1) to receive 3 immunizations every 2 weeks with DC pulsed with autologous heat-inactivated HIV-1 (Cases, n=24) or with non-pulsed DC (Controls, n=12). Changes in viral load (VL) as well as changes in CD4 cell counts have been evaluated. Additionally HIV specific responses were measured in PBMC samples from different time-points by LPR and by IFN-γ-Elispot against gag, nef and gp41 HIV overlapping peptide pools, respectively.

## Results

VL rebounded to detectable level in all the patients. At week 12 and 24, a decrease of VL >= 1 log was observed in 12/22 (55%) vs 1/11(9%) and in 7/20(35%) vs 0/10(0%) in cases and controls, respectively (p=0.02, p=0.03). CD4 drop to baseline value before any cART without differences between groups. Although only transient positive responses to HIV p24 were observed, the median change in LPR to HIV p24 at week 24 from baseline was 0.96 vs -0.50 (p=0.02) in cases vs controls, respectively. Baseline median values of the total sum of HIV specific responses against HIV peptide pools were similar in both arms (2625 versus 2283 SFC/106 PBMC, p=0.462). After vaccination, the median change of total sum of SFC/106 PBMC at week 24 was 3567 vs 838 SFC/106 PBMC (p=0.0459) in cases and controls, respectively. This difference was more evident when analyzing responses against gag p17 and Nef peptide pools (p=0.0288 and p=0.03615, respectively). No statistically significant correlations between immune responses and VL were found.

## Conclusion

These results indicate that HIV-1 specific immune responses elicited by therapeutic DC vaccines could significantly change pVL set-point after cART interruption in chronic HIV-1 infected patients.

